# Supplementary material for: How useful are body mass index and history of diabetes in COVID-19 risk stratification?
Source: PLoS One. 2022 Apr 7;17(4):e0265473. doi: 10.1371/journal.pone.0265473 (PMC8989225; doi:10.1371/journal.pone.0265473)
Supplement: Attachment — Submitted filename: Response to Reviewers.docx [file pone.0265473.s001.docx]

We thank the reviewers for the opportunity to revise our manuscript entitled **“How useful are body mass index and history of diabetes in COVID-19 risk stratification?”** We think that following the incorporation of the reviewers’ suggestions, the overall quality of the manuscript has improved greatly. Please find below our point-by-point responses to each reviewer comment. The changes detailed below can be found in the manuscript document.

| Reviewer Number, Comment Number | Comment | Response | Page Number |
| --- | --- | --- | --- |
| 1, 1 | Please provide a summary of the demographic and clinical data of the sample. This is important to evaluate the generalizability of the results. | Table 1 is now embedded in the manuscript, which includes a summary of the demographic and clinical data of the sample. | 9-10 |
| 1, 2 | Although measures race/ethnicity, income/socioeconomic status, and smoking history are mentioned in the methods, I see no mention of them anywhere else in the results. Please comment on the influence of these factors on disease severity and mortality in your sample. | Thank you for this comment, Table 1 has been embedded into the manuscript and includes a summary of participants’ race/ethnicities, income/socioeconomic status and smoking history. | 9-10 |
| 2, 1 | Overall the introduction needs to be re-worked to discuss the current literature on the relationship between COVID-19 outcomes, obesity, age, and diabetes more clearly as in its current form it reads ambiguously. | We updated references based on research published since the submission. Although there has been considerably more research published on the relationship between COVID-19, obesity and diabetes, this literature does not invalidate the statements made in the introduction. We have also added language to clarify the aim of the study to examine the value of risk stratification by BMI and diabetes diagnosis in clinical settings. | 4 |
| 2, 2 | Paragraph 1 – reference 1 does not seem to be relevant as it is a commentary without primary data and does not address the question of obesity as the sentence it is cited in does – I would consider changing this sentence so the reference is relevant or removing it altogether | Thank you for noting this discrepancy, we have removed reference 1. | 4 |
| 2, 3 | Paragraph 1 – reference 12 does not discuss obesity, only discussing fasting blood glucose – it may be better used elsewhere | Thank you for catching this difference, we have removed reference 12. | 4 |
| 2, 4 | Paragraph 1 – sentence 2 – the authors mention hyperlipidemia but this disease is not mentioned elsewhere in the manuscript at all and does not make sense in the context of the paragraph. I wonder if this was meant to state increased BMI instead. If it was referring to hyperlipidemia specifically, it should be further expounded upon. | Thank you for this comment. We have edited the text in line 40 to remove reference to hyperlipidemia.  “Several reports have suggested that excess weight is associated with COVID-19 progression, yet it is not entirely clear whether the association between obesity and adverse outcomes is due to excess weight *per se*, or whether conditions oft-associated with obesity are driving these relationships.” | 4 |
| 2, 5 | Paragraph 1 – reference 13 – the content of this reference (diabetes and lung function) is not explicitly discussed in the paragraph – may consider either adding content or removing this reference. | Reference 13 has been removed. | 4 |
| 2, 6 | Paragraph 1 – reference 17 – this reference does not seem relevant as it is related to fasting glucose and pancreatic cancer, is a negative study, and does not mention COVID-19, and is not specifically brought up in the manuscript text | Reference 17 has been removed. | 4 |
| 2, 7 | Paragraph 1 – reference 17 – the content of this reference (obesity and lung function) is not explicitly discussed in the paragraph – may consider either adding content or removing this reference. | Reference 17 has been removed. | 4 |
| 2, 8 | Paragraph 1 – reference 19 – the content of this reference (pulmonary thrombosis in COVID-19) is not discussed - may consider either adding content or removing this reference. | Reference 19 has been removed. | 4 |
| 2, 9 | Paragraph 1 – The final two sentences regarding the difference between scientific pursuits and clinical care are of unclear utility – I am not sure what the point of these are in context of the paper especially since these concepts are never referred to again in the manuscript. | Thank you for giving us the opportunity to further explain our rationale. We wanted to clarify that the scope of this paper is not to investigate underlying mechanisms accounting for the relationship between COVID-19, diabetes and obesity, but rather to evaluate the clinical utility of information readily available to frontline workers in risk stratification. This idea is reintroduced in the conclusion to cast the importance of these findings in the context of clinical care. | 4, 17 |
| 2, 10 | Demographics – the authors state ‘median household income 200% below the federal poverty line’ – I believe what is meant is ‘median household income below 200% of the federal poverty line’ | Thank you so much for catching this error, we have edited the text in line 85 to reflect this change.  “The low-income category was defined based on zip-code with a median household income below 200% of the federal poverty line.” | 7 |
| 2, 11 | Body Mass Index – it is stated that last known height and weight were used – authors should clarify if these are prior to COVID diagnosis or after. | We now clarify in lines 92-93 that “BMI was calculated from patients’ last known objectively assessed height and weight recorded in their EHR, which could have been either prior to- or after COVID-19 diagnoses.” | 6 |
| 2, 12 | Body Mass Index – reference 10 doesn’t need to be cited as using baseline as a comparator group is standard | Thank you for this suggestion, we have removed reference 10. | 6 |
| 2, 13 | Analytic Plan – reference 25 does not specify why cut point of 65 is used, only that is frequently used. | Thank you for this comment, we agree with the reviewer that we opted to choose a cut point of 65 similar studies and health services research have used this cut point. We acknowledge the arbitrary nature of our decision in the discussion, and that different cut points may yield slightly different findings. Nevertheless, because the cut point of 65 years is widely used, we believe it allows comparability with other studies. | 8, 17 |
| 2, 14 | Overall, results section is somewhat confusing to follow, going from age <65 cohort and causal mediation to age >65 cohort and mortality associations, then looking at the interactions between BMI and diabetes by age. It may be clearer to separate this section into patient characteristics first, then univariate associations, then multivariate associations (interactions between BMI, diabetes, and age), then finally causal mediation. This may help the important findings of the paper read more clearly. | Thank you for giving us the opportunity to restructure the way results are presented. To improve readability and ease of interpretation, we now separate the results into three sections: patient characteristics, models and causal mediation. The reworked results section with tables embedded can be found on pages 9-14. | 9-14 |
| 2, 15 | Table 1 and table 2 were not available for my review and should be made available. | Tables 1 and 2 have been embedded within the manuscript. | 9-10, 12-13 |
| 2, 16 | The second paragraph of the limitations as they discuss racial/ethnic disparities is not really part of limitations and would fit better in the discussion section. | Thank you for this suggestion, we have moved the second paragraph of the limitations to the end of the discussion section. | 16 |
| 2, 17 | FIGURE 1 AND 2 None of the strata have an OR of 1 – which group is the reference group in this setting that the rest are being compared to? | Thank you for this comment. We now include the reference group (BMI 18.5-24.9) in the figures for the sake of visual clarity. | N/A |
| Editor, 1 | Please ensure that your manuscript meets PLOS ONE's style requirements, including those for file naming. | The manuscript has been reformatted to meet PLOS ONE’s style requirements. | all |
| Editor, 2 | Please include your tables as part of your main manuscript and remove the individual files | Tables are now embedded within the body of the manuscript. | 9-10, 12-13 |
| Editor, 3 | Please provide additional details regarding participant consent. | Thank you for catching this omission. We have now included a sentence detailing how participant consent was obtained in lines 75-76.  “Patients provided electronic informed consent via the secure, HIPAA- compliant data capturing tool, REDCap.” | 5 |
| Editor, 4 | Please provide an amended statement that declares *all* the funding or sources of support (whether external or internal to your organization) received during this study… | The funding statement has been edited to reflect that the funding sources listed are all the sources of support received for the study. | Cover letter |
| Editor, 5 | Please state what role the funders took in the study. | The funding statement has been edited to reflect that the funders had no role in the study. | Cover letter |
| Editor, 6 | We note that you have provided funding information that is not currently declared in your Funding Statement. However, funding information should not appear in the Acknowledgments section or other areas of your manuscript. We will only publish funding information present in the Funding Statement section of the online submission form. | There is no reference to funding sources in the acknowledgements section of the manuscript. | Cover letter |
| Editor, 7 | We note that you have indicated that data from this study are available upon request. PLOS only allows data to be available upon request if there are legal or ethical restrictions on sharing data publicly. | We removed the statement that data will be available upon request. The data is available on The Immunology Database and Analysis Portal (ImmPort) as required by the National Cancer Institute, SeroNet. Private data and pre-release data are stored in private workspaces of investigators at the ImmPort site located at <http://immport.niaid.nih.gov>. Once shared, studies may be searched at the Shared Data site: [www.immport.org.](http://www.immport.org) | N/A |
| Editor, 8 | We note that you have included the phrase “data not shown” in your manuscript. Unfortunately, this does not meet our data sharing requirements. PLOS does not permit references to inaccessible data. We require that authors provide all relevant data within the paper, Supporting Information files, or in an acceptable, public repository. Please add a citation to support this phrase or upload the data that corresponds with these findings to a stable repository (such as Figshare or Dryad) and provide and URLs, DOIs, or accession numbers that may be used to access these data. Or, if the data are not a core part of the research being presented in your study, we ask that you remove the phrase that refers to these data. | We now include a table with the data that was previously not shown. See Table 3 on page 14. | 14 |
